# How is Self-Rated Health Related to the Social Network in Frail/Pre-Frail Older Adults: A Prospective Cross-Sectional Study Assessing Healthy Aging in Three Less Privileged Urban Neighborhoods

**DOI:** 10.1177/07334648251407438

**Published:** 2025-12-27

**Authors:** Cedrik Barth, Marie Bolster, Denis Gerstorf, Raphael Kohl, Dagmar Haase, Abhijit Visaria, Paul Gellert, Wolfram J. Herrmann

**Affiliations:** 1 Institute of General Practice and Family Medicine, 14903 Charité - Universitätsmedizin Berlin, Berlin, Germany; 2 Department of Psychology, 9373 Humboldt-Universität zu Berlin, Berlin, Germany; 3 Socio-Economic Panel, Deutsches Institut für Wirtschaftsforschung (DIW), Berlin, Germany; 4 Institute of Medical Sociology and Rehabilitation Science, 14903 Charité - Universitätsmedizin Berlin, Berlin, Germany; 5 Geography Department, 9373 Humboldt-Universität zu Berlin, Berlin, Germany; 6 Department of Computational Landscape Ecology, Helmholtz Centre for Environmental Research - UFZ, Leipzig, Germany; 7 Independent Researcher, Bangkok, Thailand

**Keywords:** self-rated health, frailty, social network, older adults, urban health, healthy aging

## Abstract

This prospective cross-sectional study examined whether a beneficial social network can buffer the negative association between frailty/pre-frailty and self-rated health among older adults in three less privileged urban neighborhoods. The final sample of 561 Berlin residents aged ≥65 (mean age 75.56 ± 6.66 years, 62.4% female) was assessed using the Frailty Phenotype and the Lubben Social Network Scale. Confounder-adjusted linear regression analyses were performed. The analyses showed that a strong social network is associated with better subjective health for non-frail and pre-frail older adults, but this buffering effect was not observed among frail individuals. We conclude that strengthening social networks may be beneficial for the subjective health of non-frail and pre-frail older adults. This buffering effect may diminish with advanced physiological limitations.

**What This Paper Adds**

• Our study suggests that the protective effect of a strong social network on subjective health may not be universal. Instead, it may be limited to only non-frail and pre-frail older adults.
• Our findings emphasize the importance of considering the complex interplay between physical and social factors in relation to the subjective health of older adults.
• By focusing on older adults in less privileged urban neighborhoods, our work contributes a perspective on healthy aging in a specific socioeconomic context.

**Applications of Study Findings**

• From a practical perspective, our results suggest that interventions to strengthen social networks should specifically target non-frail and pre-frail older adults.
• Primary care providers may proactively screen for individuals who might benefit from social interventions.
• Future research is needed, particularly randomized controlled trials, to determine whether social network interventions effectively improve the subjective health of robust and pre-frail older adults.

## Introduction

The ongoing demographic shift and urbanization lead to a growing number of older adults in urban areas, making healthy aging an increasingly important challenge. This is all the more important considering that worldwide, the number of individuals aged 65 and above is projected to reach approximately two billion by 2050 (Gibson & Jung, 2006; Kinsella & Phillips, 2005). The World Health Organization defines healthy aging as a process that promotes well-being in older age (Rudnicka et al., 2020). One domain of healthy aging is the general health status, which is often measured by self-rated health (Lu et al., 2019). Thus, achieving and sustaining good self-rated health is important for healthy aging. Self-rated health reflects existing illnesses and functional limitations, as well as an individual’s perception of well-being (Wu et al., 2013). It integrates both physical and psychological dimensions of health and has been shown to be a valid indicator of future health service use and mortality (Berger et al., 2015; DeSalvo et al., 2006; Idler & Benyamini, 1997; Van Der Linde et al., 2013), even when objective health parameters are included (Idler & Benyamini, 1997).

However, achieving and sustaining good self-rated health is greatly affected by physical decline, particularly by frailty. According to Fried et al.'s Frailty Phenotype (Fried et al., 2001), frailty is defined as a clinical syndrome in which three or more of the following five criteria must be present: weight loss, weakness, exhaustion, slowness, and low physical activity. For an individual to be classified as pre-frail, one or two of the five specified criteria must be met (Fried et al., 2001). According to the findings from a systematic review from 2021, the prevalence of frailty among those aged ≥50 across 62 countries, including the Fried Phenotype model, was 12% and pre-frailty was 46% (O’Caoimh et al., 2021). Furthermore, 26.1% of people aged 85 and over were frail in 2012 (Collard et al., 2012). In line with this, several studies have shown a positive association between frailty and poor self-rated health (Chu et al., 2021; Huohvanainen et al., 2016; Mello et al., 2014).

Given the detrimental impact of frailty on health outcomes such as self-rated health, coupled with its substantial prevalence and anticipated rise due to the demographic shift, identifying modifiable buffers is crucial. According to the stress-buffering hypothesis, specific factors can moderate the association between a stressor (such as frailty) and negative outcomes (such as poor self-rated health) (Cohen & Wills, 1985). The social network, for example, could fulfill this role, as it has been shown to improve health through multiple mechanisms (Thoits, 2011), and it is important for self-rated health and healthy aging (Berkman, 1995; Dai et al., 2016; Muramatsu et al., 2010). Several constructs of social factors have been identified in the literature, and it has been demonstrated that social network size and related social factors, such as social support and relationships, play a significant role in the development of frailty and subsequent health outcomes for those affected (Barghouth et al., 2024; Mehrabi & Béland, 2024). As a weak social network indicates a risk for social isolation (Lubben et al., 2006), it is crucial to develop effective measures to combat this problem. Various interventions against social isolation have been developed and it is recommended to target specific groups of older adults (Fakoya et al., 2020). Furthermore, social interventions could be particularly beneficial for older people (Bellelli et al., 2023). This suggests that targeting frail or pre-frail older adults specifically could be a compelling model. Regarding the modifiability of social networks, intervention studies have shown that, for example, the neighborhood social network of older adults can be expanded (Harada et al., 2021). At an individual healthcare level, care models such as social prescribing are a promising approach (Herrmann & Napierala, 2024; Napierala et al., 2022). In line with the stress-buffering hypothesis and given its established role and modifiability, explicitly testing the social network’s potential to buffer the effects of frailty on self-rated health could be a viable strategy.

However, there is still limited evidence regarding this particular interaction. Orlandini et al. assessed the relationship between the Frailty Index and the Social Vulnerability Index, comprising 30 self-reported items from various social domains, on self-rated health (Orlandini et al., 2024). Nonetheless, evidence is still limited as to whether the social network can modify the adverse effects of frailty (Mehrabi & Béland, 2020; Roppolo et al., 2016).

Although the positive association between the social network and health is well-established, few studies have explicitly tested its protective effect, especially for frail or pre-frail older adults in vulnerable urban populations. Thus, the hypothesis of this study is that a beneficial social network can buffer the negative association between frailty/pre-frailty and self-rated health among older adults in three less privileged neighborhoods in Berlin.

## Methods

### Study Design, Study Site, and Study Participants

This paper analyzes data from the Ageing Well Study. This cohort study examines the health and social needs of older adults in urban areas of Berlin, Germany. The questionnaire covers topics such as health and health care utilization, neighborhoods, loneliness and social contacts, mobility, and sociodemographic characteristics.

This analysis is based on the baseline interviews of the first wave in Berlin. The study protocol has been published elsewhere (Bolster et al., 2025).

A random sample of 5,250 people aged 65 and over in three medium to low socioeconomic districts of Berlin were invited to participate in the study. Of these, 561 gave written informed consent, and a standardized Computer-Assisted Personal Interview was conducted by trained interviewers in the participants’ homes between December 2023 and June 2024. This consisted of a questionnaire and functional tests, including grip strength and walking speed measurements.

Inclusion criteria were: age ≥65 years, official residency in a selected neighborhood, and providing written informed consent. Exclusion criteria were: cognitive or linguistic inability to understand the German questionnaire and written consent, and having a legal guardian.

### Measurement Variables

#### Self-Rated Health

Self-rated health was measured using the single question: ''In general, would you describe your health as excellent, very good, good, fair or poor?'' and used as a continuous variable (Jürges et al., 2008).

#### Frailty

Frailty was assessed using the Fried et al. Frailty Phenotype, yielding a score from 0 to 5 where 0 indicates robustness, 1 or 2 indicates pre-frailty, and 3 or more indicates frailty (Fried et al., 2001). We used this concept to detect frailty because it is the most commonly used measure for frailty (Bouillon et al., 2013). The five criteria were:

Unintentional weight loss (>5 kg in the last year). Weakness: grip strength was tested based on the average value from two measurements taken on the dominant hand, using the Saehan SH5001 hand dynamometer. Low grip strength cut-offs were based on German normative data (men: ≤29 kg; women: ≤18 kg) (Huemer et al., 2023). Exhaustion: two of the Center for Epidemiologic Studies–Depression scale (CES-D) questions were used (Orme et al., 1986). During the last week: (a) I had the feeling that everything was exhausting and (b) I couldn’t “get going.” Participants were classified as exhausted if they answered “often” to at least one of these questions. Slowness: participants walked 2.5 m twice at normal speed (using a walking aid, if needed), the required times were measured and averaged. The cut-off value (≤0.8 m/s) for the walking speed average was taken from the latest consensus of the European Working Group on Sarcopenia in Older People 2 (Cruz-Jentoft et al., 2019). Lastly, low physical activity was assessed using the World Health Organization’s Global Physical Activity Questionnaire. Less than 600 metabolic equivalent minutes per week was considered low activity (Armstrong & Bull, 2006).

#### Social Network

The Lubben Social Network Scale-6 (LSNS-6) was used to assess the social network. It evaluates both kin and non-kin relationships, measuring network size, perceived availability of support, and intimacy of ties. The LSNS-6 ranges from 0 to 30, with higher scores indicating the most supportive and strongest social network. Besides, the LSNS-6 is also used to indicate whether a person is at risk for social isolation. Scores <12 points indicate this (Lubben et al., 2006). Despite this risk classification, however, the LSNS-6 was used as a continuous variable in the analyses.

#### Confounders

Prior to the data analysis, potential confounding factors were assessed.

Sociodemographic variables included age, gender based on the Diversity Minimal Item Set (Stadler et al., 2023), and Subjective Social Status (SSS), measured using the MacArthur Scale (ranging from 1 = lowest to 10 = highest) (MacArthur Scale of Subjective Social Status–Adult Version|SPARQtools, o. J). SSS was chosen over objective Socioeconomic Status (SES) indicators because it has been shown to be an independent predictor of poorer health, even when SES is taken into account (Hoebel et al., 2015). Clinical control variables included the number of chronic conditions (lifetime) based on a list of 23 different conditions, the Global Activity Limitation Indicator (GALI) (Jagger et al., 2010) and depression, as determined by the two depression-related questions of the Patient Health Questionnaire-4 (PHQ-4) (Kroenke et al., 2009).

### Statistical Analyses

To assess the interplay between frailty and social network on self-rated health, a linear regression analysis with an interaction term was conducted. All models contained self-rated health as the dependent variable, with one corresponding to poor and five to excellent self-rated health. Model 1 included both the frailty category and the LSNS-6 score as independent variables. In Model 2 the interaction term frailty * LSNS-6 was added. Model 3 also included the confounders age, gender, and MacArthur Scale. In addition, it was further controlled for chronic conditions, the GALI and depression in Model 4. Missing values were deleted listwise. For sensitivity, we performed an additional regression where missing frailty criteria were imputed by assigning one point each as if the cut-off was met. Regression results were reported as the unstandardized estimate (beta) with the 95% confidence interval (CI). Unstandardized estimates were chosen for the main tables to enable the effect size to be interpreted directly in the original unit of self-rated health. Additionally, a sensitivity analysis was conducted using standardized coefficients. R version 4.4.2 was used for all analyses (R Core Team, 2024).

## Results

### Sample Characteristics

At baseline, 561 participants were included. Table 1 presents sample characteristics stratified by frailty category. Mean age was 75.56 ± 6.66 years and about two-thirds of the participants were female (62.4%).

**Table 1.** Baseline Characteristics of the Study Population Stratified by Frailty Categories

Variable	Category	Robust	Pre-frail	Frail	Total
		*n* = 83	*n* = 298	*n* = 104	*N* = 561
**Self-rated health (SRH)** (one = poor - five = excellent)					
SRH mean (SD)		3.13 (0.81)	2.77 (0.86)	2.04 (0.72)	2.61 (0.91)
SRH-categories *n* (%)	Poor	1 (1.2)	15 (5.0)	23 (22.1)	56 (10.0)
	Fair	15 (18.1)	97 (32.6)	56 (53.9)	198 (35.3)
	Good	43 (51.8)	140 (47.0)	23 (22.1)	230 (41.0)
	Very good	20 (24.1)	35 (11.7)	2 (1.9)	62 (11.1)
	Excellent	4 (4.8)	11 (3.7)	-	15 (2.7)
SRH missings *n* (%)					-
**Frailty**					
Frailty mean (SD)		0.00 (0.00)	1.45 (0.50)	3.32 (0.58)	1.60 (1.14)
Frailty-categories *n* (%)	Frail	-	-	104 (100.0)	104 (18.5)
	Pre-frail	-	298 (100.0)	-	298 (53.1)
	Robust	83 (100.0)	-	-	83 (14.8)
Frailty missings *n* (%)					76 (13.6)
**Social network**					
LSNS-6 mean (SD)		17.95 (5.91)	16.19 (6.07)	12.87 (5.75)	15.58 (6.21)
LSNS-6 risk for isolation	Yes	10 (12.0)	69 (23.4)	43 (41.3)	149 (26.6)
*n* (%)	No	73 (88.0)	226 (76.6)	61 (58.7)	406 (72.4)
LSNS-6 missings *n* (%)					6 (1.1)
**Confounders**					
Age mean (SD)		72.55 (4.99)	74.80 (6.20)	79.18 (5.95)	75.56 (6.66)
Age group *n* (%)	65–69	27 (32.5)	60 (20.8)	6 (5.8)	123 (21.9)
	70–74	30 (36.1)	94 (32.6)	16 (15.5)	155 (27.6)
	75–79	15 (18.1)	53 (18.4)	26 (25.2)	107 (19.1)
	80–84	11 (13.3)	59 (20.5)	36 (35.0)	119 (21.2)
	≥85	-	22 (7.6)	19 (18.4)	57 (10.2)
Age missings *n* (%)					-
Gender *n* (%)	Female	53 (63.9)	185 (62.1)	68 (65.4)	350 (62.4)
Gender missings *n* (%)					-
MacArthur SSS mean (SD)		6.67 (1.44)	6.35 (1.74)	5.86 (1.40)	6.30 (1.66)
MacArthur SSS missings *n* (%)					6 (1.1)
Chronic cond. mean (SD)		3.93 (2.08)	4.36 (2.60)	5.90 (2.88)	4.62 (2.64)
Chronic cond. missings *n* (%)					-
GALI *n* (%)	Severely	12 (14.8)	48 (16.3)	44 (42.7)	132 (24.0)
	A little	13 (16.0)	89 (30.3)	33 (32.0)	159 (28.9)
	Not	56 (69.1)	157 (53.4)	26 (25.2)	259 (47.1)
GALI missings *n* (%)					11 (2.0)
Depression	Yes	-	19 (6.5)	23 (23.0)	47 (8.6)
*n* (%)	No	82 (100.0)	275 (93.5)	77 (77.0)	501 (91.4)
Depression missings *n* (%)					13 (2.3)

SRH = self-rated health; SD = standard deviation; SSS = subjective social status; cond. = conditions; LSNS-6 = the lubben social network scale-6.

Figure 1 displays additional distribution of self-rated health, frailty, LSNS-6, and age.

**Figure 1.**
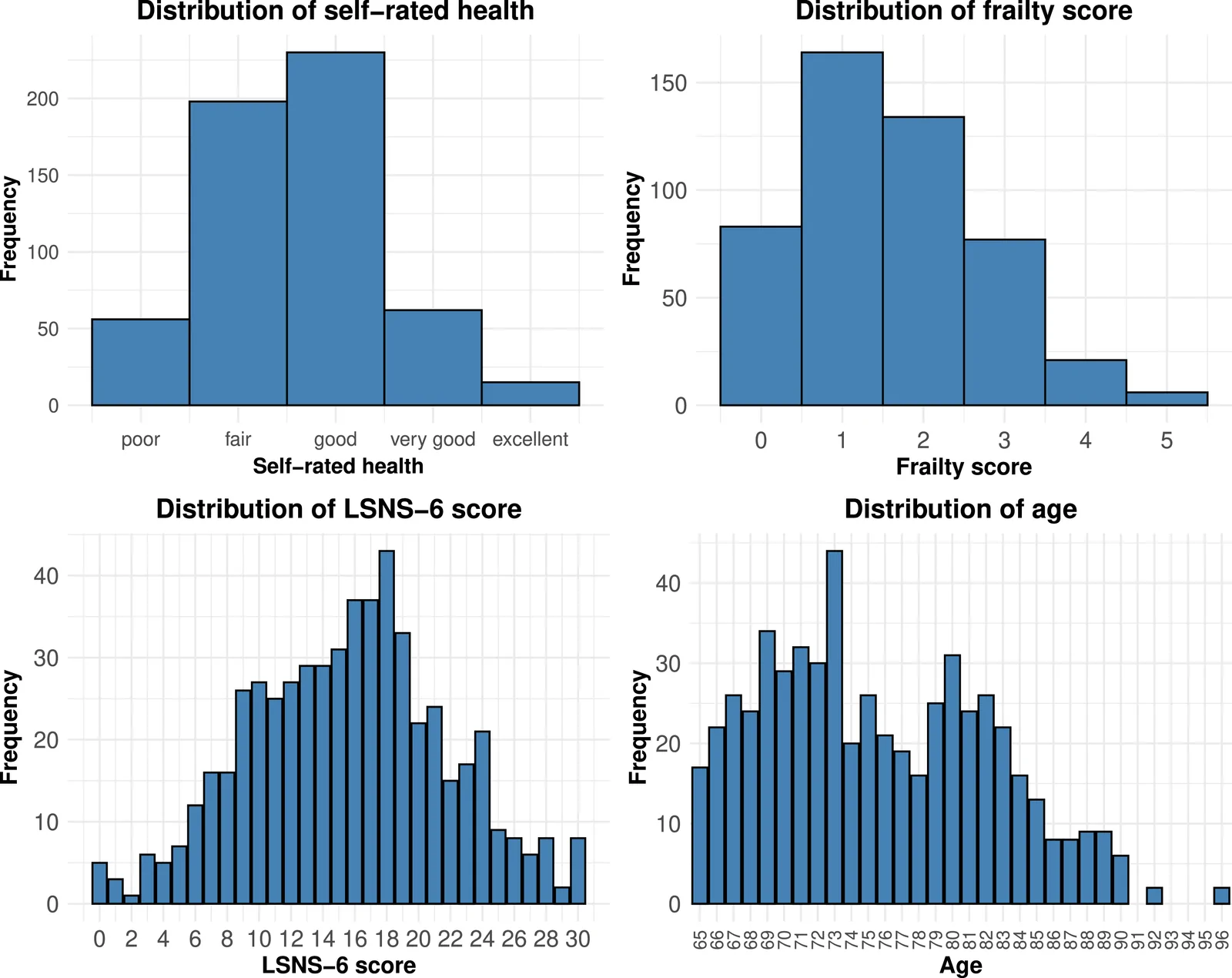
Distribution of self-rated health, frailty, social network score, and age in the sample. Self-rated health and age *N* = 561; Frailty *n* = 485; LSNS-6 *n* = 555. LSNS-6 = the lubben social network scale-6

#### Characteristics by Frailty Categories

Most participants were pre-frail *n* = 298 (53.1%) and frailty was higher in older age groups. More than 50% of the frail people were ≥80 years old, and no one aged ≥85 was considered robust. While most robust and pre-frail participants reported good self-rated health, the frail group mostly had fair self-rated health.

Social networks were reduced in pre-frail and frail people. In the sample, pre-frail participants were more than twice as likely, and frail participants more than three times as likely, to be at risk for social isolation. Compared to those not at risk for social isolation, those at risk were more than twice as likely to be frail (16.9% vs. 35.2%). Tables showing baseline characteristics stratified by self-rated health and social isolation can be found in Appendix A.

### Correlation of Frailty, Self-Rated Health, and LSNS-6

The Pearson correlation between the frailty score and self-rated health was −0.44 (95%CI: −0.51, −0.36), between self-rated health and LSNS-6 was 0.32 (95%CI: 0.24, 0.39) and between frailty score and LSNS-6 was −0.31 (95% CI: −0.39, −0.23). Figure 2 depicts the relationship between the frailty categories, self-rated health, and LSNS-6, showing that participants with a higher LSNS-6 score tend to report a better self-rated health (see Appendix B for a frailty score version).

**Figure 2.**
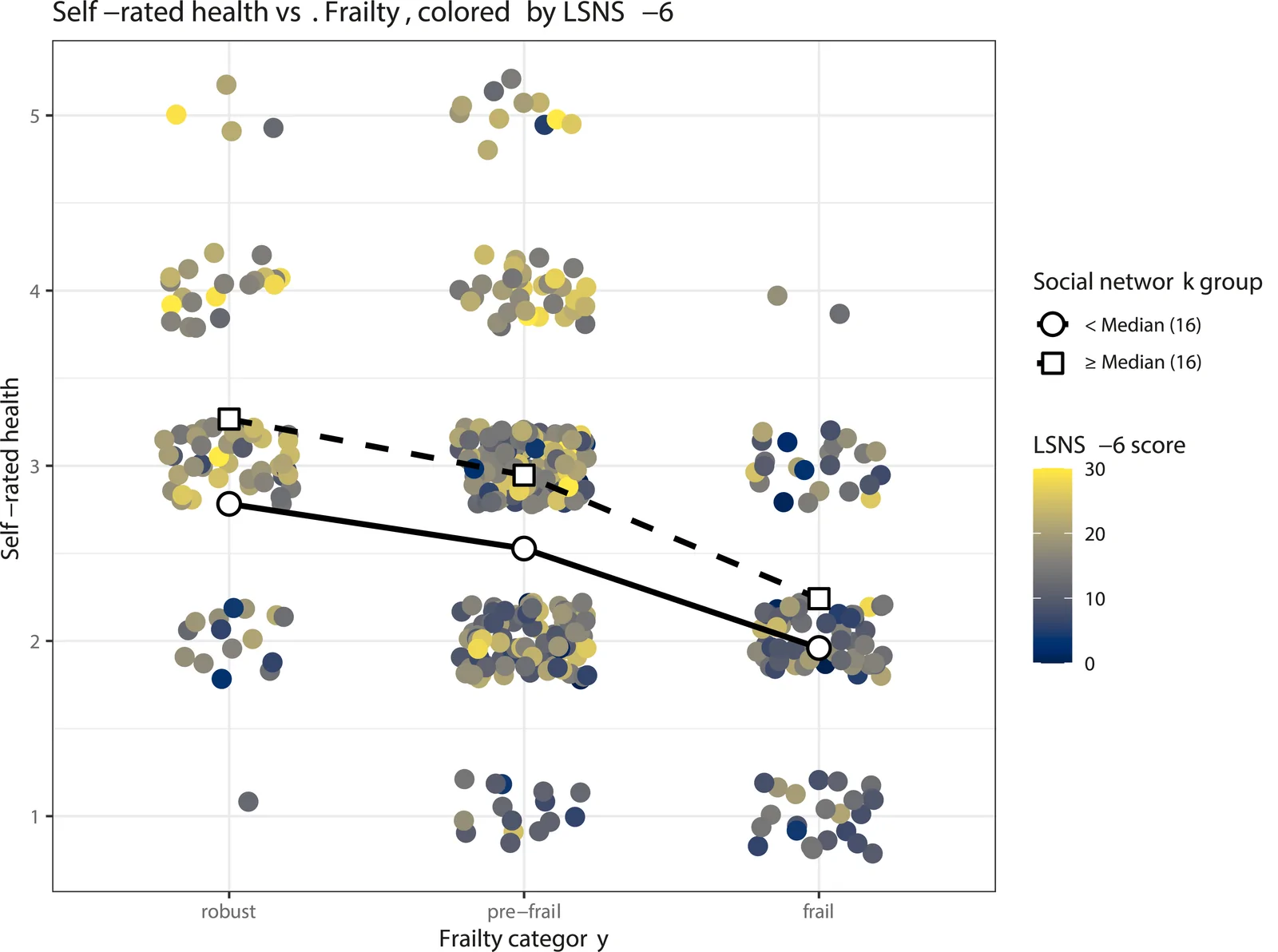
Relationship between self-rated health, frailty categories, and the social network in the sample. *n* = 482

### Linear Regression Analysis

Table 2 presents the regression results. Model 4, while including the interaction term and controlling for confounders, has the best Akaike Information Criterion (AIC) and the highest adjusted R^2^. In the following, we focus on Model 4.

**Table 2.** Linear Regression Results on Self-Rated Health

Variables	Model 1 Estimate (95% CI)	Model 2 Estimate (95% CI)	Model 3 Estimate (95% CI)	Model 4 Estimate (95% CI)
Dependent variable = self-rated health (one = poor-five = excellent)				

Intercept	2.455	2.264	2.572	3.407
	(2.180, 2.729)	(1.714, 2.814)	(1.544, 3.600)	(2.446, 4.368)
1. Pre-frail	−0.300**	−0.177	−0.106	0.023
	(−0.495, −0.105)	(−0.785, 0.431)	(−0.710, 0.498)	(−0.535, 0.581)
2. Frail	−0.902***	−0.424	−0.304	0.068
	(−1.140, −0.664)	(−1.109, 0.242)	(−0.972, 0.364)	(−0.559, 0.695)
3. LSNS-6	0.038***	0.048**	0.043**	0.043**
	(0.026, 0.050)	(0.019, 0.078)	(0.014, 0.072)	(0.017, 0.070)
4. Age			−0.011	−0.006
			(−0.022, 0.001)	(−0.017, 0.005)
5. Gender (male)			−0.021	−0.094
			(−0.167, 0.124)	(−0.231, 0.042)
6. MacArthur scale			0.085***	0.025
			(0.039, 0.130)	(−0.018, 0.069)
7. Chronic conditions				−0.089***
				(−0.116, −0.062)
8. GALI				−0.253***
				(−0.342, −0.163)
9. Depression (yes)				−0.234
				(−0.480, 0.012)
10. Pre-frail * LSNS-6		−0.006	−0.008	−0.011
		(−0.039, 0.026)	(−0.041, 0.024)	(−0.041, 0.018)
11. Frail * LSNS-6		−0.033	−0.034	−0.037*
		(−0.072, 0.007)	(−0.073, 0.006)	(−0.073, −0.000)
adjusted R^2^	0.218	0.220	0.242	0.371
AIC	1,152.33	1,152.80	1,140.67	1,022.94
*p*-value	<0.001	<0.001	<0.001	<0.001

Significance level: *p* < 0.1, *p < 0.05, **p < 0.01, ***p < 0.001.

Model 1 + 2 are based on *n* = 482 observations: 79 observations deleted as missing.

Model 3 is based on *n* = 481 observations: 80 observations deleted as missing.

Model 4 is based on *n* = 466 observations: 95 observations deleted as missing.

For frail older adults a change in LSNS-6 from the first to the third quartile (Q1 = 11, Q3 = 20) could result in a higher self-rated health of 0.054, for pre-frail of 0.288 and for robust of 0.387. In detail, for frail participants, it is estimated that the self-rated health could be increased by 0.006 (95% CI: −0.018, 0.031) per one-point LSNS-6 increase, for pre-frail participants by 0.032 (95% CI: 0.017, 0.046) and for robust participants by 0.043 (95% CI: 0.017, 0.070).

Figure 3 visualizes this interaction as an interaction plot of Model 4: In general, self-rated health is predicted to be lower with weaker social networks and more frailty. The interaction plot shows that for robust and pre-frail individuals, the social network may make a difference. Those with a stronger social network have better self-rated health than those with a weaker one. When frailty is present, the predicted self-rated health remains poor regardless of the LSNS-6 score.

**Figure 3.**
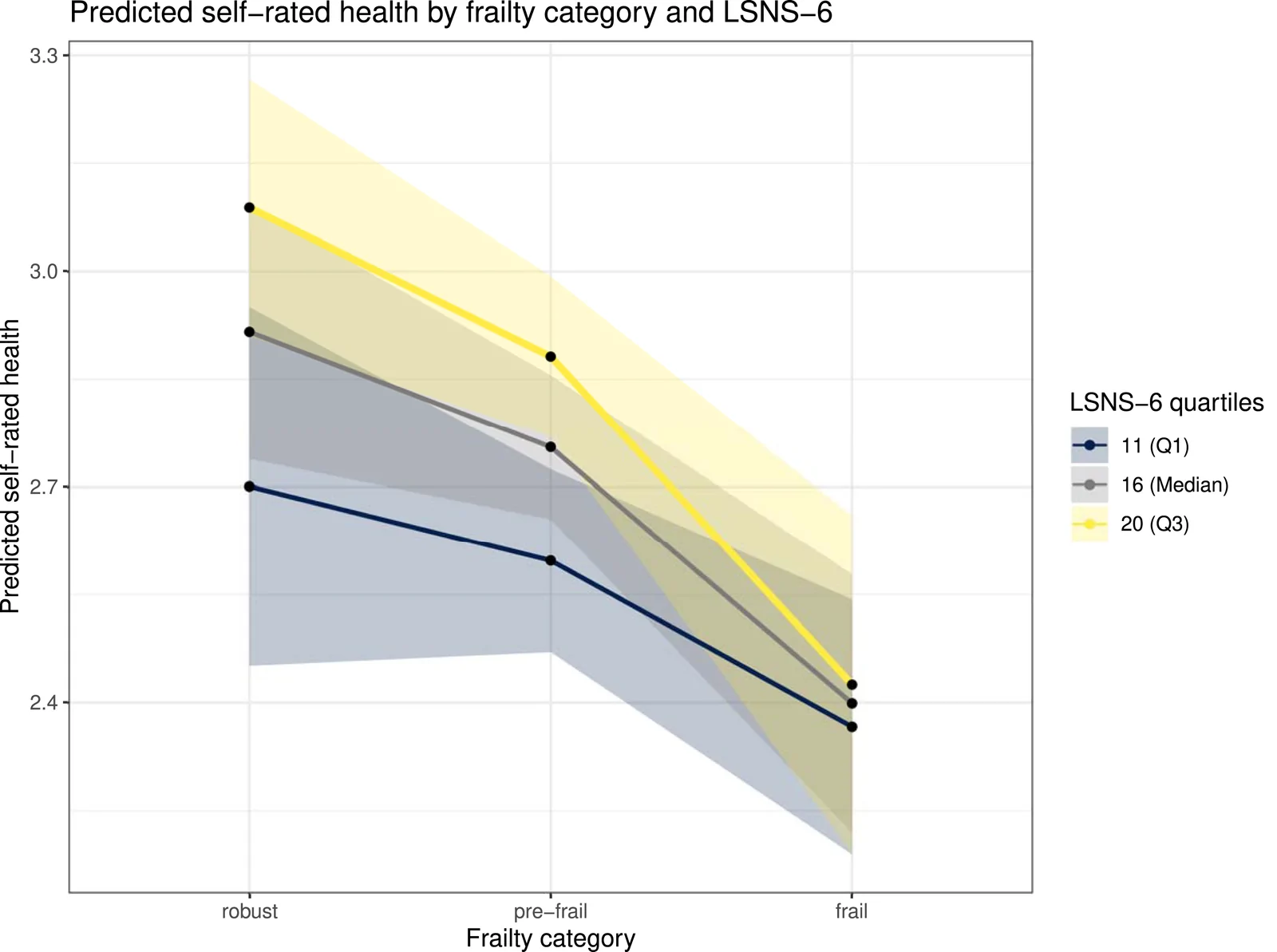
Interaction plot of the final regression model. The lines represent the predicted self-rated health for individuals at the 25th (Q1 = 11), 50th (Median = 16), and 75th (Q3 = 20) percentiles of LSNS-6. Surrounding the lines are the 95% confidence intervals. The independent variables age, MacArthur Scale, chronic conditions, and the GALI are set to their mean values. Gender is female and depression is set to no. *n* = 466

### Missing Values and Sensitivity Analysis

For the final regression model (Model 4) 95 participants (16.9%) were excluded from the analysis. In total, 76 people were excluded because at least one of the five frailty criteria could not be assessed. Of these, 44 people were unable to complete the walking speed assessment, 23 participants were unable to complete a single grip strength measurement on either hand and there was missing data for physical activity (14), weight loss (12), and exhaustion (six). Additionally, there was missing data for depression (13), the GALI (11), LSNS-6 (six) and the MacArthur scale (six). For the sensitivity analysis, where single imputation was used instead of listwise deletion, as described in section “2.3. Statistical analyses,” we re-included the 76 participants with missing frailty criteria. This resulted in an additional 29 pre-frail and 47 frail participants. The analysis showed consistent results (see Appendix C).

The same results were reached when a regression analysis was conducted with a frailty score ranging from zero to five. Model 4 “Score” then showed an interaction term with an estimate of −0.011 (95% CI: −0.021, −0.001) (table and interaction plot in Appendix D). Setting gender or depression to different reference groups did not alter the interaction plot. Subgroup linear regression analysis showed that the social network did not have a significant effect on self-rated health in frail people. However, it had a significant effect on robust and pre-frail people (table in Appendix E). For comparison, allowing the assessment of the relative strength of each predictor, a regression table containing a standardized self-rated health, social network, age, MacArthur Scale, and chronic conditions variable can be found in Appendix F. The Standardized coefficients show that the effect of the social network on self-rated health is comparable in size to the effects of chronic conditions, the GALI, and depression.

## Discussion

### Summary of Key Findings

This cross-sectional study investigated whether a beneficial social network could buffer the negative effect of frailty/pre-frailty on self-rated health among older adults living across three less privileged neighborhoods in Berlin. Our results revealed that both frailty and social network are independently associated with self-rated health. The regression analysis and the interaction plot showed that among non-frail and pre-frail participants, a higher LSNS-6 score was associated with better self-rated health. Contrary to our hypothesis, the self-rated health of frail individuals may be poor regardless of their social network strength.

### Interpretation of Results

Our findings align with prior research linking frailty and poor self-rated health (Chu et al., 2021; Huohvanainen et al., 2016; Mello et al., 2014), and with studies demonstrating that social network strength and other related social factors, correlate with self-rated health (Berkman, 1995; Dai et al., 2016). Furthermore, Barghouth et al. demonstrated that older adults in Berlin with poorer social resources were twice as likely to become frail (Barghouth et al., 2024). Our results also showed that people at risk for social isolation were twice as likely to be frail compared to those not at risk.

Mehrabi et al.'s scoping review revealed that only four studies had examined the interaction effects of frailty and social isolation on health (Mehrabi & Béland, 2020). For instance, they reported a significant interaction term of frailty and social isolation with regard to disability, resulting in a higher level of disability in isolated than in non-isolated frail Italian older adults (Roppolo et al., 2016). However, only a few studies have investigated whether social isolation or the social network could modify frailty’s adverse effects. Evidence suggests that social factors, such as having a strong social network, can enhance cognitive health and reduce depressive symptoms in older, frail adults (Mehrabi & Béland, 2024). Yet, their interplay with frailty is rarely assessed. To our knowledge, only the study by Orlandini et al. assessed the relationship between frailty and social vulnerability, comprising 30 self-reported items from various social domains, on self-rated health (Orlandini et al., 2024). In a further sensitivity analysis, they included an interaction term, revealing that very high values of the Frailty Index or the Social Vulnerability Index significantly reduced the effect of the other. This aligns with our finding that a strong social network is associated with better self-rated health among non-frail and pre-frail participants, and that the self-rated health of frail individuals may be poor regardless of their social network strength. Furthermore, we demonstrated that when both frailty and the social network score are high, self-rated health remains poor, as would be expected with a high frailty value.

However, compared to our study, Orlandini et al. used different constructs, namely, the Frailty Index and the Social Vulnerability Index, covering multiple social domains. In contrast, our study, using the LSNS-6, shows that the social network in particular could be a target for improving subjective health.

We have shown that for robust and pre-frail individuals, beneficial social networks may improve self-rated health, partly according to our buffer hypothesis. One reason for this might be that a stronger social network contributes to subjective health and healthy aging by enabling older adults to handle daily stressors (Muramatsu et al., 2010). This emphasizes the need for interventional studies to assess programs enhancing social networks for robust and pre-frail older adults.

Regarding frailty there are multiple factors that could explain why we had to reject our buffer hypothesis. Firstly, frailty may be so restrictive that a beneficial social network can barely offset its negative impact on self-rated health. Secondly, frailty itself may lead to social withdrawal, explaining why the social network cannot mitigate its effects. Finally, maintaining a strong social network may require physical resources, as well as the emotional and social resources it provides. For instance, many frail older people may find it difficult to leave the house, which can make maintaining a strong network more challenging. Thus, it may not improve frail individuals’ self-rated health, as they may be unable to provide resources that preserve the social network.

### Strengths and Limitations

Strengths of this study are its prospective design and the random sampling in three particular neighborhoods. Conducting the interviews in participants’ homes or nursing homes increased the likelihood of older adults with limited mobility participating.

However, particularly vulnerable older adults, such as those who were very old, sick, had dementia, or who did not speak German, were less able to participate. Additionally, we only focused on the urban environment. This may prevent the results from being generalized to a broader population of older adults.

Another limitation is that, although the LSNS-6 effectively measures network size, perceived availability of support and intimacy of ties, it does not capture other comprehensive qualitative dimensions of social relationships. Furthermore, although the final regression model controlled for depression, its prevalence may have affected the frailty assessment. For instance, it may have resulted in the exhaustion criteria being applied when it is more likely to be due to depression than to frailty. Additionally, chronic conditions were based on lifetime diagnoses and did not provide a validated measurement tool. Lastly, some participants were interviewed simultaneously because they lived together, which may have influenced their answers.

### Recommendation

From a practical perspective, our results suggest that strengthening social networks could effectively improve the self-rated health of robust and pre-frail older adults. As Fakoya et al. state in their review of social isolation interventions, many different interventions have been tested, but there is no general approach to combatting social isolation. Hence, future research should target specific groups of older adults (Fakoya et al., 2020). Our findings suggest that especially robust and pre-frail individuals could be targeted. This could be achieved at two levels. Firstly, at a community level, where for example neighborhood measures could be employed, as interventional studies have shown that the neighborhood social network of older adults can be expanded. This could thereby have a positive impact on health (Harada et al., 2021). Secondly, at an individual healthcare level, it has been shown that social prescribing, for example, is a promising approach to combating psychosocial problems (Napierala et al., 2022) and that German general practitioners view services like social prescribing as a meaningful addition to primary care settings (Herrmann & Napierala, 2024). However, further research, such as randomized controlled trials, is needed to determine whether social network interventions positively impact subjective health of robust and pre-frail older adults. Although social networks alone cannot offset the negative impact of frailty on self-rated health, strengthening them and thereby reducing the risk for social isolation remains essential.

### Conclusion and Implications

In conclusion, our study shows the complex interplay of frailty and the social network on subjective health. It reveals that for non-frail and pre-frail older adults, a strong social network is associated with better subjective health. Thus, strengthening social networks among these individuals could be beneficial. This can be done through both community-based initiatives and individual-level healthcare interventions. However, this buffering effect was not observed among frail older adults, suggesting that the influence of social networks on subjective health may be diminished in the context of advanced physiological limitations.

Our results have clear implications for general practice and research prioritization. The high standardized coefficient for the social network suggests a strong connection to self-rated health, comparable in magnitude to that of chronic conditions or depression. Thus, we recommend that primary care providers should be aware of their older patients’ social networks. Additionally, we strongly recommend conducting a randomized controlled trial to test if the subjective health of pre-frail and robust older adults can be significantly improved by social interventions targeting the social network.

## Supplemental Material

Supplemental Material—How is Self-Rated Health Related to the Social Network in Frail/Pre-Frail Older Adults: A Prospective Cross-Sectional Study Assessing Healthy Aging in Three Less Privileged Urban Neighborhoods Supplemental Material for How is Self-Rated Health Related to the Social Network in Frail/Pre-Frail Older Adults: A Prospective Cross-Sectional Study Assessing Healthy Aging in Three Less Privileged Urban Neighborhoods by Cedrik Barth, Marie Bolster, Denis Gerstorf, Raphael Kohl, Dagmar Haase, Abhijit Visaria, Paul Gellert, and Wolfram J. Herrmann in Journal of Applied Gerontology

## Data Availability

The data underlying this article will be shared on reasonable request to the corresponding author. However, it will only be shared in part, as the written consent included an opt-in/opt-out option and some participants chose not to have their data included in the open data set.*
