# Supplementary material for: How is Self-Rated Health Related to the Social Network in Frail/Pre-Frail Older Adults: A Prospective Cross-Sectional Study Assessing Healthy Aging in Three Less Privileged Urban Neighborhoods
Source: J Appl Gerontol. 2025 Dec 27;45(12):2438–49. doi: 10.1177/07334648251407438 (PMC13740172; doi:10.1177/07334648251407438)
Supplement: Supplementary file 1 [file sj-pdf-1-jag-10.1177_07334648251407438.pdf]

Supplementary Material of:

How is self-rated health related to the social network in frail/pre-frail older adults: A prospective cross-sectional study assessing healthy aging in three less privileged urban neighborhoods

Contents

Appendix A:

Table A.1 - Table of the baseline characteristics stratified by self-rated health.

Table A.2 - Table of the baseline characteristics stratified by social isolation.

Appendix B:

Fig. B.1 - Raw data plot with frailty as a score.

Appendix C:

Table C.1 - Regression results with all frailty missings set to “point for Frailty”.

Fig. C.1 - Interaction Plot with all frailty missings set to “point for Frailty”.

Appendix D:

Table D.1 - Regressions results with frailty as a score.

Fig. D.1 - Interaction Plot with frailty as a score.

Appendix E:

Table E.1 - Regression table with frailty subgroups.

Appendix F:

Table F.1 - Regression table with standardized (mean = 0, SD = 1) coefficients.

Appendix A:

Table A.1 - Table of the baseline characteristics stratified by self-rated health.

Variable	Category	Excellent n = 15 (2.7%)	Very good n = 62 (11.1%)	Good n = 230 (41.0%)	Fair n = 198 (35.3%)	Poor n = 56 (10%)	Total N = 561
Self-rated health (SRH) (one=poor - five=excellent):							
**SRH** Mean (SD)							2.61 (0.91)
**SRH** Missings n (%)							-
Frailty:							
**Frailty** Mean (SD)		0.93 (0.70)	0.88 (0.80)	1.32 (0.96)	1.98 (1.12)	2.79 (1.24)	1.60 (1.14)
**Frailty - catego-ries** n (%)	Frail	-	2 (3.5)	23 (11.2)	56 (33.3)	23 (59.0)	104 (18.5)
	Pre-frail	11 (73.3)	35 (61.4)	140 (68.0)	97 (57.7)	15 (38.5)	298 (53.1)
	Robust	4 (26.7)	20 (35.1)	43 (20.9)	15 (8.9)	1 (2.6)	83 (14.8)
**Frailty** Missings n (%)							76 (13.6)
Social network:							
**LSNS-6** Mean (SD)		20.00 (6.68)	19.58 (5.51)	16.11 (5.91)	14.29 (5.89)	12.31 (6.00)	15.58 (6.21)
**LSNS-6 risk for isolation** n (%)	Yes	1 (6.7)	2 (3.2)	50 (22.0)	66 (33.7)	30 (54.5)	149 (26.6)
	No	14 (93.3)	60 (96.8)	177 (78.0)	130 (66.3)	25 (45.5)	406 (72.4)
**LSNS-6** Missings n (%)							6 (1.1)
Confounders:							
**Age** Mean (SD)		70.93 (5.64)	72.98 (6.28)	75.28 (6.52)	76.88 (6.60)	76.09 (6.83)	75.56 (6.66)

**Age group** n (%)	65 - 69	6 (42.9)	14 (25.9)	50 (22.0)	26 (13.3)	10 (18.5)	123 (21.9)
	70 - 74	5 (35.7)	18 (33.3)	72 (31.7)	47 (24.1)	13 (24.1)	155 (27.6)
	75 - 79	2 (14.3)	10 (18.5)	36 (15.9)	47 (24.1)	12 (22.2)	107 (19.1)
	80 - 84	-	9 (16.7)	50 (22.0)	49 (25.1)	11 (20.4)	119 (21.2)
	≥85	1 (7.1)	3 (5.6)	19 (8.4)	26 (13.3)	8 (14.8)	57 (10.2)
**Age** Missings n (%)							-
**Gender** n (%)	Female	7 (46.7)	39 (62.9)	149 (64.8)	127 (64.1)	28 (50.0)	350 (62.4)
**Gender** Missings n (%)							-
**MacArthur SSS** Mean (SD)		7.53 (1.81)	6.98 (1.70)	6.37 (1.51)	6.14 (1.68)	5.52 (1.62)	6.30 (1.66)
**MacArthur SSS** Missings n (%)							6 (1.1)
**Chronic conditions** Mean (SD)		2.00 (1.69)	3.02 (1.77)	3.99 (2.43)	5.58 (2.59)	6.29 (2.43)	4.62 (2.64)
**Chronic cond.** Missings n (%)							-
**GALI** n (%)	Severely	-	7 (11.7)	32 (14.3)	54 (27.6)	39 (70.9)	132 (23.5)
	A little	2 (13.3)	10 (16.7)	58 (25.9)	77 (39.3)	12 (21.8)	159 (28.3)
	Not	13 (86.7)	43 (71.7)	134 (59.8)	65 (33.2)	4 (7.3)	259 (46.2)
**GALI** Missings n (%)							11 (2.0)
**Depres-sion** n (%)	Yes	-	2 (3.2)	7 (3.1)	24 (12.5)	14 (26.4)	47 (8.4)
	No	15 (100.0)	60 (96.8)	219 (96.9)	168 (87.5)	39 (73.6)	501 (89.3)
**Depression** Missings n (%)							13 (2.3)

SRH = Self-rated health, SD = Standard deviation, SSS = Subjective Social Status

Table A.2 - Table of the baseline characteristics stratified by social isolation.

Variable	Category	No risk for Social Isolation	At risk for Social Isolation	Total N = 561
Social network:				
**LSNS-6** Mean (SD)		18.42 (4.43)	7.85 (2.87)	15.58 (6.21)
**LSNS-6 risk for isolation** n (%)	Yes	-	149 (26.8)	149 (26.6)
	No	406 (73.2)	-	406 (72.4)
**LSNS-6** Missings n (%)				6 (1.1)
Self-rated health (SRH) (one=poor - five=excellent):				
**SRH** Mean (SD)		2.77 (0.90)	2.18 (0.79)	2.61 (0.91)
**SRH - Categories** n (%)	Poor	25 (6.2)	30 (20.1)	56 (10.0)
	Fair	130 (32.0)	66 (44.3)	198 (35.3)
	Good	177 (43.6)	50 (33.6)	230 (41.0)
	Very good	60 (14.8)	2 (1.3)	62 (11.1)
	Excellent	14 (3.4)	1 (0.7)	15 (2.7)
**SRH** Missings n (%)				-
Frailty:				
**Frailty** Mean (SD)		1.43 (1.10)	2.10 (1.15)	1.60 (1.14)
**Frailty - categories** n (%)	Frail	61 (16.9)	43 (35.2)	104 (18.5)
	Pre-frail	226 (62.8)	69 (56.6)	298 (53.1)
	Robust	73 (20.3)	10 (8.2)	83 (14.8)
**Frailty** Missings n (%)				76 (13.6)
Confounders:				

**Age** Mean (SD)		75.00 (6.44)	77.11 (7.06)	75.56 (6.66)
**Age group** n (%)	65 - 69	80 (20.5)	25 (17.0)	123 (21.9)
	70 - 74	119 (30.4)	33 (22.4)	155 (27.6)
	75 - 79	76 (19.4)	30 (20.4)	107 (19.1)
	80 - 84	84 (21.5)	34 (23.1)	119 (21.2)
	≥85	32 (8.2)	25 (17.0)	57 (10.2)
**Age** Missings n (%)				-
**Gender** n (%)	Female	262 (64.5)	84 (56.4)	350 (62.4)
**Gender** Missings n (%)				-
**MacArthur SSS** Mean (SD)		6.55 (1.60)	5.63 (1.65)	6.30 (1.66)
**MacArthur SSS** Missings n (%)				6 (1.1)
**Chronic conditions** Mean (SD)		4.41 (2.61)	5.21 (2.68)	4.62 (2.64)
**Chronic cond.** Missings n (%)				-
**GALI** n (%)	Severely	80 (20.1)	50 (34.0)	132 (23.5)
	A little	112 (28.1)	46 (31.3)	159 (28.3)
	Not	206 (51.8)	51 (34.7)	259 (46.2)
**GALI** Missings n (%)				11 (2.0)
**Depression** n (**%**)	Yes	19 (4.8)	27 (18.8)	47 (8.4)
	No	381 (95.2)	117 (81.2)	501 (89.3)
**Depression** Missings n (%)				13 (2.3)

SRH = Self-rated health, SD = Standard deviation, SSS = Subjective Social Status

Appendix B:

Fig. B.1 - Raw data plot with frailty as a score.

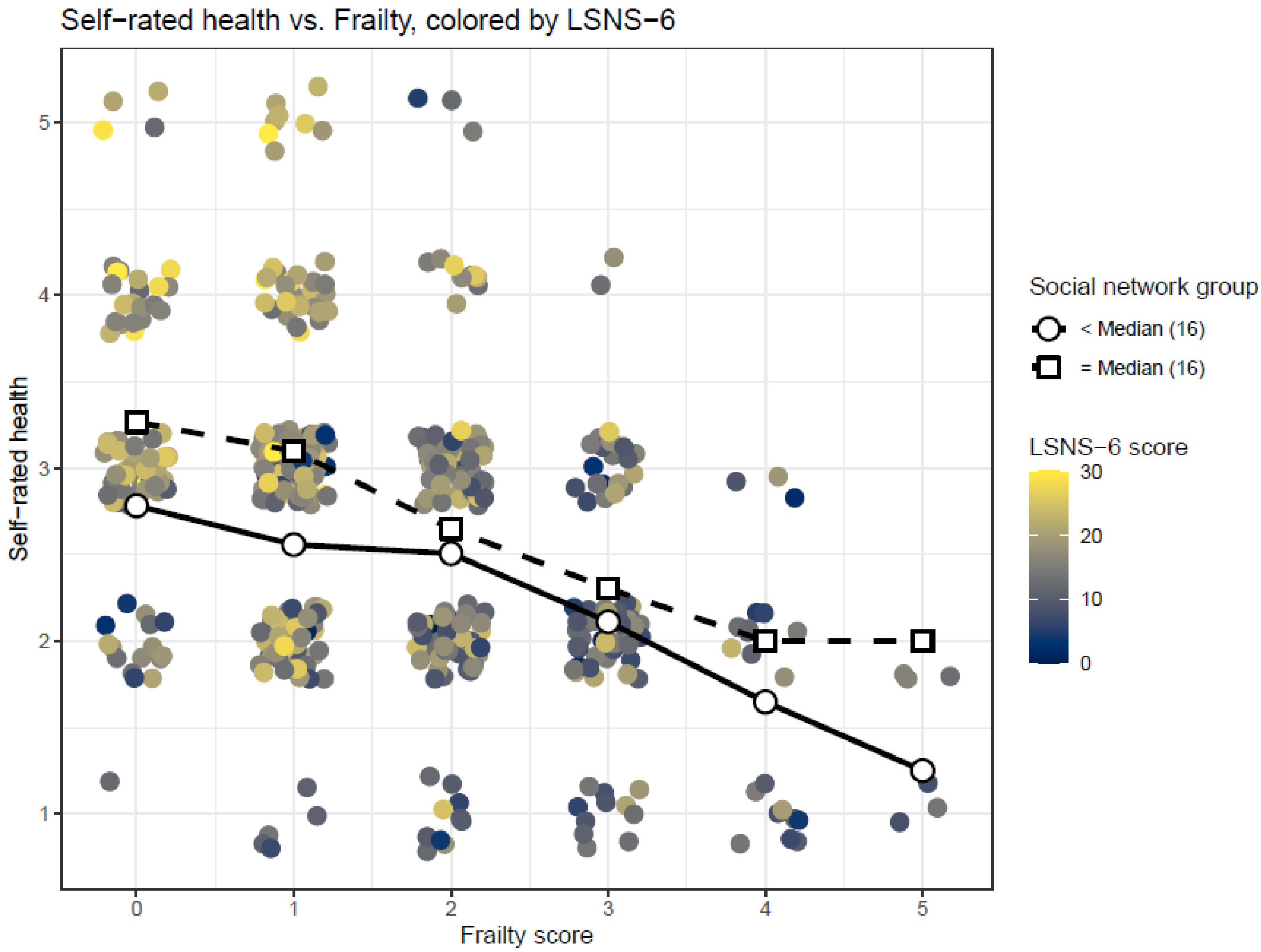

n = 482

Appendix C:

Table C.1 - Regression results with all frailty missings set to "point for Frailty".

Variables	Sens. Model 1 Estimate (95%CI)	Sens. Model 2 Estimate (95%CI)	Sens. Model 3 Estimate (95%CI)	Sens. Model 4 Estimate (95%CI)
Dependent variable = Self-rated health (one=poor - five=excellent)				
Intercept	2.559<br>(2.292, 2.827)	2.264<br>(1.709, 2.819)	2.361<br>(1.403, 3.319)	3.316<br>(2.417, 4.214)
1. Pre-frail	-0.332 ***<br>(-0.528, -0.137)	-0.190<br>(-0.800, 0.420)	-0.140<br>(-0.744, 0.465)	0.020<br>(-0.538, 0.578)

2. Frail	-0.927 *** (-1.152, -0.703)	-0.244 (-0.880, 0.391)	-0.242 (-0.879, 0.396)	0.109 (-0.485, 0.704)
3. LSNS-6	0.032 *** (0.021, 0.043)	0.048 ** (0.019, 0.078)	0.044 ** (0.015, 0.073)	0.044 ** (0.018, 0.071)
4. Age			-0.006 (-0.017, 0.004)	-0.004 (-0.014, 0.006)
5. Gender (male)			-0.028 (-0.165, 0.109)	-0.108 . (-0.236, 0.020)
6. MacArthur Scale			0.068 ** (0.026, 0.110)	0.012 (-0.029, 0.052)
7. Chronic conditions				-0.084 *** (-0.109, -0.058)
8. GALI				-0.285 *** (-0.368, -0.202)
9. Depression (Yes)				-0.220 . (-0.455, 0.014)
10. Pre-frail * LSNS-6		-0.007 (-0.040, 0.026)	-0.009 (-0.041, 0.024)	-0.013 (-0.043, 0.016)
11. Frail * LSNS-6		-0.047 * (-0.083, -0.010)	-0.042 * (-0.078, -0.005)	-0.042 * (-0.076, -0.008)
adjusted R^2	0.209	0.220	0.236	0.368
AIC	1342.48	1336.30	1312.04	1175.77
p-value	< 0.001	< 0.001	< 0.001	< 0.001

*Significance level: . $p < 0.1$, * $p < 0.05$, ** $p < 0.01$, *** $p < 0.001$*

Sens. Model 1+2 are based on n = 555 observations: 6 observations deleted as missing
Sens. Model 3 is based on n = 550 observations: 11 observations deleted as missing
Sens. Model 4 is based on n = 533 observations: 28 observations deleted as missing
Sens. = Sensitivity

Fig. C.1 - Interaction Plot with all frailty missings set to "point for Frailty".

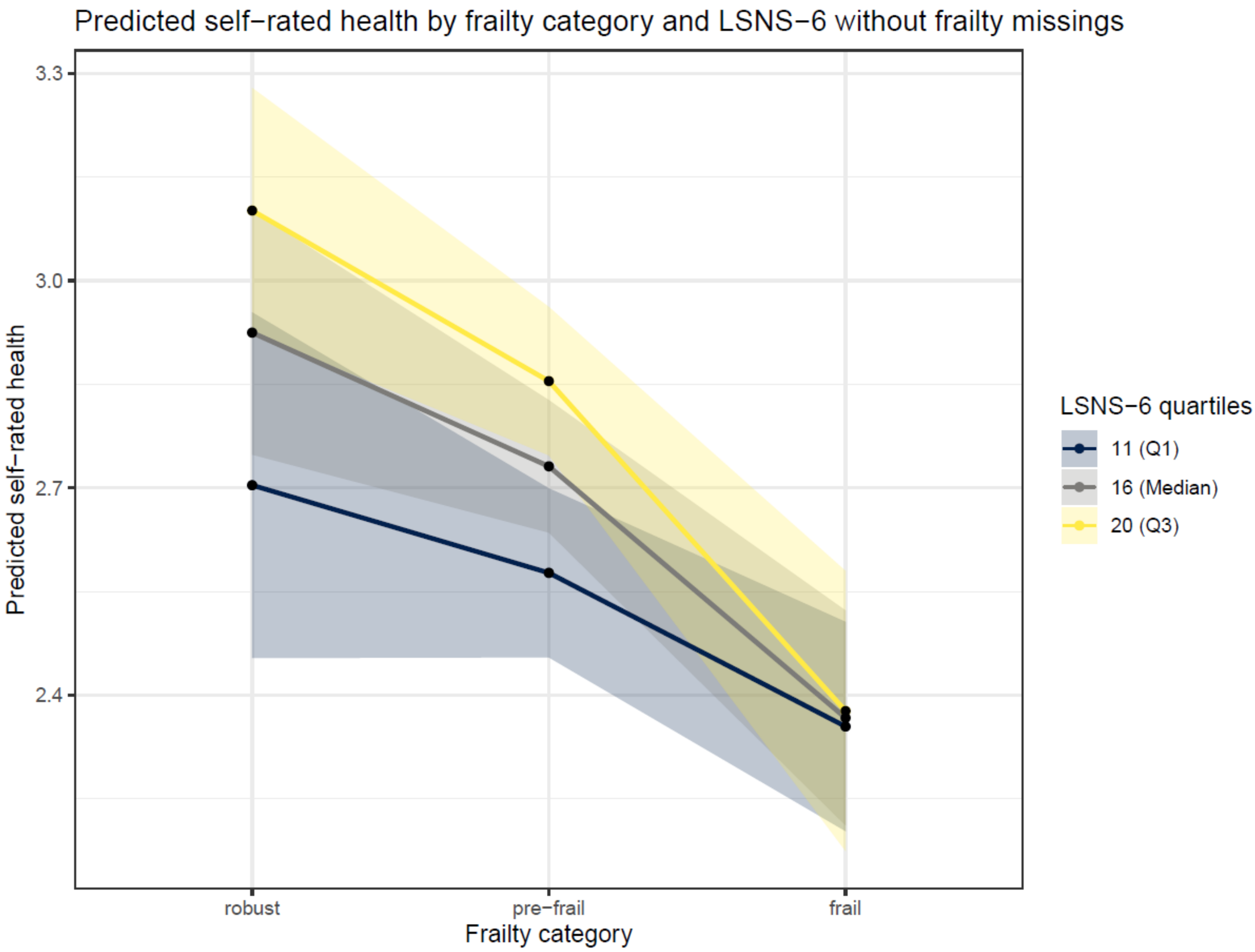

The lines represent the predicted self-rated health for individuals at the 25th (Q1 = 11), 50th (Median = 16), and 75th (Q3 = 20) percentiles of LSNS-6. Surrounding the lines are the 95% confidence intervals. The independent variables age, MacArthur Scale, chronic conditions and the GALI are set to their mean values. Gender is female and depression is set to no. n = 531

Appendix D:

Table D.1 - Regressions results with frailty as a score.

Variables	Model 1 Score Estimate (95%CI)	Model 2 Score Estimate (95%CI)	Model 3 Score Estimate (95%CI)	Model 4 Score Estimate (95%CI)
Dependent variable = Self-rated health (one=poor - five=excellent)				
Intercept	2.597<br>(2.345, 2.848)	2.411<br>(2.053, 2.770)	2.576<br>(1.634, 3.517)	3.342<br>(2.452, 4.233)
1. Frailty score	-0.287 ***<br>(-0.351, -0.222)	-0.175 *<br>(-0.342, -0.009)	-0.147 .<br>(-0.314, 0.020)	0.023<br>(-0.138, 0.184)

2. LSNS-6	0.034 ***<br>(0.022, 0.046)	0.044 ***<br>(0.026, 0.065)	0.040 ***<br>(0.020, 0.060)	0.044 ***<br>(0.026, 0.063)
3. Age			-0.008<br>(-0.020, 0.004)	-0.005<br>(-0.016, 0.006)
4. Gender (male)			-0.032<br>(-0.176, 0.112)	-0.101<br>(-0.236, 0.035)
5. MacArthur Scale			0.079 ***<br>(0.035, 0.124)	0.024<br>(-0.020, 0.067)
6. Chronic conditions				-0.088 ***<br>(-0.114, -0.061)
7. GALI				-0.248 ***<br>(-0.338, -0.158)
8. Depression (Yes)				-0.235 .<br>(-0.481, 0.012)
9. Frailty score * LSNS-6		-0.008<br>(-0.018, 0.003)	-0.008<br>(-0.018, 0.003)	-0.011 *<br>(-0.021, -0.001)
adjusted R^2	0.239	0.241	0.258	0.376
AIC	1137.88	1137.84	1128.34	1017.42
p-value	< 0.001	< 0.001	< 0.001	< 0.001

*Significance level: . $p < 0.1$, * $p < 0.05$, ** $p < 0.01$, *** $p < 0.001$*

Model 1+2 Score are based on n = 482 observations: 79 observations deleted as missing
Model 3 Score is based on n = 481 observations: 80 observations deleted as missing
Model 4 Score is based on n = 466 observations: 95 observations deleted as missing

Fig. D.1 - Interaction Plot with frailty as a score.

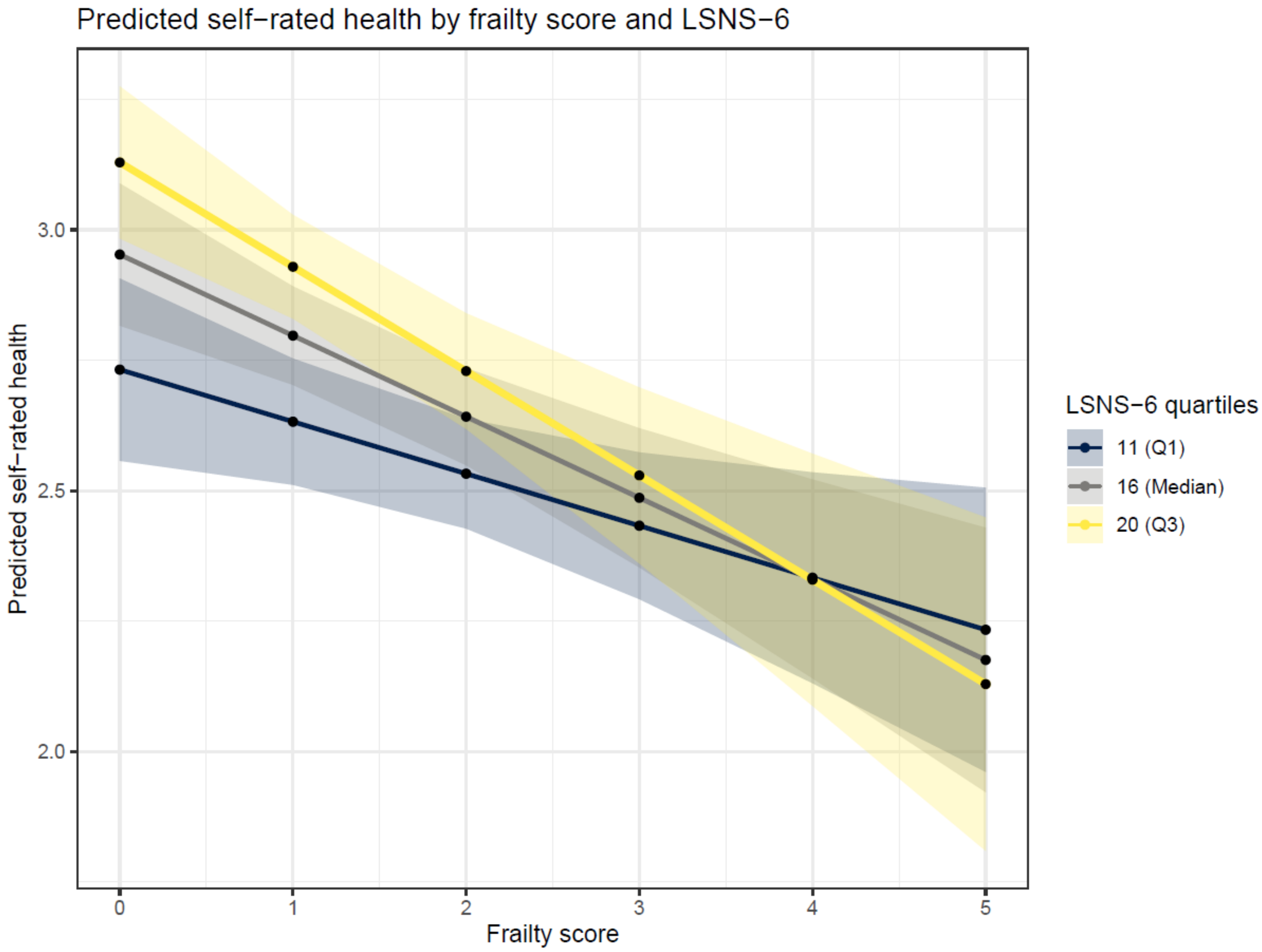

The lines represent the predicted self-rated health for individuals at the 25th (Q1 = 11), 50th (Median = 16), and 75th (Q3 = 20) percentiles of LSNS-6. Surrounding the lines are the 95% confidence intervals. The independent variables age, MacArthur Scale, chronic conditions and the GALI are set to their mean values. Gender is female and depression is set to no. n = 466

Appendix E:

Table E.1 - Regression table with frailty subgroups.

Variables	Model Robust Estimate (95%CI)	Model Pre-frail Estimate (95%CI)	Model Frail Estimate (95%CI)
Dependent variable = Self-rated health (one=poor - five=excellent)			
Intercept	4.237<br>(1.761, 6.713)	3.891<br>(2.785, 4.996)	1.983<br>(0.040, 3.926)
1. LSNS-6	0.040 **<br>(0.011, 0.069)	0.031 ***<br>(0.016, 0.045)	0.014<br>(-0.010, 0.037)

2. Age	-0.028<br>(-0.062, 0.007)	-0.009<br>(-0.022, 0.005)	0.011<br>(-0.012, 0.037)
3. Gender (male)	-0.049<br>(-0.399, 0.301)	-0.173 .<br>(-0.349, 0.002)	0.045<br>(-0.235, 0.325)
4. MacArthur Scale	0.108 .<br>(-0.015, 0.231)	0.005<br>(-0.048, 0.058)	-0.012<br>(-0.117, 0.093)
5. Chronic conditions	-0.066<br>(-0.148, 0.017)	-0.125 ***<br>(-0.159, -0.090)	-0.021<br>(-0.070, 0.027)
6. GALI	-0.178<br>(-0.400, 0.045)	-0.228 ***<br>(-0.347, -0.110)	-0.338 ***<br>(-0.506, -0.170)
7. Depression (Yes)	*constant (No)*	-0.332 .<br>(-0.680, 0.016)	-0.253<br>(-0.582, 0.077)
adjusted R^2	0.17	0.325	0.177
AIC	189.74	631.52	206.19
p-value	0.003	< 0.001	< 0.001

*Significance level: . p < 0.1, * p < 0.05, ** p < 0.01, *** p < 0.001*

Model Robust is based on n = 80 observations: 3 observations deleted as missing
Model Pre-frail is based on n = 287 observations: 11 observations deleted as missing
Model Frail is based on n = 99 observations: 5 observations deleted as missing

Appendix F:

Table F.1 - Regression table with standardized (mean = 0, SD = 1) coefficients.

Variables	Model 1-standardized Estimate (95%CI)	Model 2-standardized Estimate (95%CI)	Model 3-standardized Estimate (95%CI)	Model 4-standardized Estimate (95%CI)
Dependent variable = Self-rated health-standardized				
Intercept	0.477<br>(0.285, 0.669)	0.449<br>(0.245, 0.653)	0.402<br>(0.188, 0.616)	0.810<br>(0.561, 1.059)
1. Pre-frail	-0.331 **<br>(-0.547, -0.116)	-0.306 **<br>(-0.534, -0.076)	-0.261 *<br>(-0.488, -0.034)	-0.172<br>(-0.384, 0.040)
2. Frail	-0.996 ***<br>(-1.259, -0.734)	-1.035 ***<br>(-1.312, -0.759)	-0.914 ***<br>(-1.200, -0.627)	-0.554 ***<br>(-0.830, -0.278)
3. LSNS-6-standardized	0.259 ***<br>(0.177, 0.341)	0.332 **<br>(0.132, 0.531)	0.295 **<br>(0.097, 0.493)	0.295 **<br>(0.114, 0.477)
4. Age-standardized			-0.078 .<br>(-0.165, 0.009)	-0.047<br>(-0.128, 0.034)
5. Gender (male)			-0.024<br>(-0.185, 0.137)	-0.104<br>(-0.255, 0.046)

6. MacArthur Scale-standardized			0.155 *** (0.072, 0.237)	0.046 (-0.033, 0.126)
7. Chronic conditions-standardized				-0.259 *** (-0.338, -0.181)
8. GALI				-0.279 *** (-0.377, -0.180)
9. Depression (Yes)				-0.258 . (-0.530, 0.013)
10. Pre-frail * LSNS-6		-0.044 (-0.269, 0.181)	-0.057 (-0.280, 0.165)	-0.079 (-0.283, 0.126)
11. Frail * LSNS-6		-0.226 (-0.497, 0.045)	-0.230 . (-0.498, 0.038)	-0.251 * (-0.499, -0.002)
adjusted R^2	0.218	0.220	0.242	0.371
AIC	1247.89	1248.36	1236.04	1115.33
p-value	< 0.001	< 0.001	< 0.001	< 0.001

*Significance level: . p < 0.1, * p < 0.05, ** p < 0.01, *** p < 0.001*

Model 1-standardized + 2-standardized are based on n = 482 observations: 79 observations deleted as missing

Model 3-standardized is based on n = 481 observations: 80 observations deleted as missing

Model 4-standardized is based on n = 466 observations: 95 observations deleted as missing

List of Abbreviations

AIC
Akaike information criterion
CES-D
Center for epidemiological studies depression
CI
Confidence interval
GALI
Global activity limitation indicator
LSNS-6
Lubben social network scale - 6
PHQ-4
Patient health questionnaire - 4
SD
Standard deviation
SES
Socioeconomic status
SRH
Self-rated health

SSS
Subjective social status.
